# A national survey of public awareness of antimicrobial resistance in Nigeria

**DOI:** 10.1186/s13756-020-00739-0

**Published:** 2020-05-20

**Authors:** Emelda E. Chukwu, David A. Oladele, Oluwatoyin B. Awoderu, Ebelechukwu E. Afocha, Rahman G. Lawal, Ismail Abdus-salam, Folasade T. Ogunsola, Rosemary A. Audu

**Affiliations:** 1 Center for Infectious Disease Research, Microbiology Department, Nigerian Institute of Medical Research, Yaba, Lagos State Nigeria; 2 Clinical Science Department, Nigerian Institute of Medical Research, Yaba, Lagos State Nigeria; 3 Epidemiology Department, Lagos State Ministry of Health, Ikeja, Lagos Sate Nigeria; 4 Department of Medical Microbiology and Parasitology, College of Medicine, University of Lagos, Akoka, Lagos State, Nigeria; 5 Center for Human Virology and Genomics, Microbiology Department, Nigerian Institute of Medical Research, Yaba, Lagos State Nigeria

**Keywords:** Antibiotics, Antimicrobial resistance, Awareness, General public, Nigeria

## Abstract

**Background:**

One of the objectives of the Global Action Plan by the World Health Organization (WHO) to contain antimicrobial resistance (AMR), is to improve global awareness through effective communication and education. Comprehensive information on the level of awareness of AMR among Nigerian public is deficient. This study was therefore designed to assess the current level of awareness and knowledge of the Nigerian public of AMR.

**Methods:**

Pre-tested and validated questionnaire was used to obtain information from the general public across the six geopolitical zones (North Central, North East, North West, South East, South South and South West) in Nigeria. Multi-stage sampling was used to select one state from each zone and respondents were selected through a multi-stage sampling technique. Responses to eight questions were used to grade the level of knowledge categorized as poor, fair and good. Collation and analysis of data were performed at the Microbiology Department of the Nigerian Institute of Medical Research (NIMR), Lagos, Nigeria, using SPSS version 24.0.

**Results:**

Questionnaires from 482 respondents comprising 242 (50.2%) females and 240 (49.8%) males from six states (Lagos, Ebonyi, Delta, Plateau, Borno and Jigawa) were analyzed. Of the 482 respondents, 322 (66.8%) had taken antibiotics in the last six months out of which 31.3% were without prescription. 26.1% of respondents believe they don’t need to complete the dosage as long as they feel better. Although 272(56.5%) of the respondents were familiar with the term “antibiotic resistance”, only 40(8.3%) had good knowledge of AMR. A majority (76.6%) believed that they were powerless to stop the spread of AMR. There was no association between the gender of respondents and knowledge of AMR (*p* = 0.13). However, respondents from Ebonyi and Delta states in southern Nigeria were more likely to have good knowledge of AMR (X^2^ = 53.22, *P* < 0.0001). The respondents in the urban area had a higher score for knowledge level compared to the rural dwellers, though this was not statistically significant within and across states.

**Conclusion:**

This survey provides an insight into the level of AMR awareness and antibiotic use in the wider Nigeria public. Our findings show that about a third of the general public consume antibiotics obtained without prescription. There is an overall poor understanding of antimicrobial resistance and/or proper use of antibiotics among respondents. It is critical that more holistic public enlightenment programs are carried out to increase awareness of AMR and promote responsible use of antibiotics.

## Introduction

Antibiotics have been used successfully to treat infections for many years and have made the management of infectious diseases easier thereby decreasing morbidity and mortality [1]. However, recent evidence suggests that the gains achieved through antibiotics are threatened by the development of antimicrobial resistance (AMR) in both hospital and community settings [2–4]. This recent trend has rendered standard treatment ineffective, complicating patient management and increasing patient morbidity and mortality [5, 6].

Globally, drug resistance causes an estimated 700,000 deaths each year. If current trends continue, it is projected that, by 2050, AMR could result in over 10 million deaths per year and over 100 trillion USD in lost output globally [4]. The World Health Organization (WHO) is coordinating a global campaign to raise awareness of antibiotic resistance and encourage best practices among the public, policy makers, health and agriculture professionals, to avoid further emergence and spread of antibiotic resistance. Part of the activities of the Global Action Plan (GAP) to tackle the growing challenge of antimicrobial resistance is to improve global awareness and understanding of AMR through effective communication, education and training [4].

Antibiotic resistance is aggravated by the misuse and overuse of antibiotics, as well as poor infection prevention and control [7]. A holistic approach to tackle the menace of AMR will involve steps taken at all levels of society (public, policy makers, health and agriculture professionals) to reduce the impact and limit the spread of resistance. The general public can play a key role by taking actions to prevent infections to avoid the need for antibiotics and using antibiotics only when prescribed by a certified health professional. Other remedial actions will include; always taking the full prescription, never using left-over antibiotics and never sharing antibiotics with others.

Resistance to specific antibiotics has been reported in different parts of Nigeria. In 2009, Ghebremedhin et al. [8], reported the emergence of a community-associated methicillin-resistant *Staphylococcus aureus* in southwest Nigeria while Lamikanra et al. [9], reported the rapid evolution of flouroquinolone-resistant *Escherichia coli* in a Nigerian community. Okesola and Oni [10] also reported high antibiotic resistance rates among common Gram-positive and Gram-negative isolates from various clinical specimens in a tertiary hospital in Nigeria. It has been reported that the abuse of antibiotics by the public is an important risk factor for antibiotic resistance [11]. A study by Igbeneghu [12] on the knowledge and practices in the use of antibiotics among a group of Nigerian university students showed a high rate of consumption of antibiotics within this group. The authors noted that they mostly obtain their antibiotics from unofficial sources without a physician’s prescription and do not complete their course of antibiotic therapy. Similarly, a survey of some communities in Jos, Nigeria, showed that inadequate antibiotic knowledge and negative attitudes towards antibiotics use exist among consumers [13].

In light of the above findings, it is clear that majority of the existing data are localized and inconclusive and more work needs to be done. At present, there is a dearth of comprehensive information about the general public’s knowledge of antimicrobial resistance at a national level which would guide initiation of strategic interventions that would tackle emergence of AMR. Therefore, this survey provides at a glance, the current public awareness and common behaviors related to antibiotics use within the Nigerian population.

## Methodology

### Study setting

The study sites were mapped based on the six geo-political zones in Nigeria (North Central, North East, North West, South East, South South and South West). Multi-stage random sampling method was used to select one state from each geopolitical zone giving a total of six states. The selected states included; Plateau State (North Central), Borno State (North East), Ebonyi State (South East), Delta State (South South), Lagos State (South West) and Jigawa State (North West). Similarly, two local government areas (LGA) were randomly selected from each state to reflect the urban and rural areas. Two Administrative Wards were selected in each LGA through random sampling. Stratified random sampling method was used to select five streets in the selected Wards and every nth house in the selected street (usually the 5th house was used because the average number of habitable houses on a street is 20). The questionnaires were administered to head of the household as well as to every member of the household that is 18 years and above and gave consent. One household per house was recruited into the study. In the event that a household head declined consent to participate in the study, another household in the same house was approached and in places where there is only one household per a house, the next house was enlisted. The study was conducted within a period of six months, from June–November 2019. Collation, processing and analysis of data were carried out at the Microbiology Department of the Nigerian Institute of Medical Research (NIMR) Yaba, Lagos, Nigeria.

### Study design/ sampling tool

This was a cross-sectional study done using a pre-tested and validated questionnaire adapted from [7] https://www.who.int/drugresistance/documents/baselinesurveynov2015/en/. The data collection tool consisted of 15 main articles, divided into three sections, targeting information on the use of antibiotics (5 questions), knowledge of antibiotics (4 questions) and knowledge of antibiotic resistance (6 questions). One question in the antibiotic resistance section asked respondents to answer true or false to eight knowledge questions and was used to grade the respondents’ knowledge with a score of one for correct answer and zero for incorrect answer. The percentage scores were determined and then used to classify respondents’ knowledge levels. Those whose scores were ≤ 25% were considered to have low antibiotics knowledge while those whose scores were ≥ 75% were considered to have good antibiotics knowledge. Respondents whose knowledge scores were between 25 and 75% were regarded to have a fair knowledge level. The questionnaire involved open ended and closed ended structured questions that were pre-tested using 30 participants randomly selected from the non-research members of the NIMR staff, specifically, administrative staff, security officers and cleaners. The result of the pilot study was analyzed, and the feedbacks were used to fine-tune, adapt and structure the data collection tool. The validated questionnaire was either self-administered or interviewer-administered depending on the level of comprehension of the respondents. In order to allow for a better and wider inclusivity, interpreters were recruited from the local communities and trained to interpret and administer the questionnaires as the need arose. Participants who clearly stated that they neither take nor are aware of what antibiotics are, where excluded from the study. The questionnaires were randomly administered to four hundred and eighty-two (482) participants from selected states.

### Statistical analysis

Quantitative data was summarized using descriptive statistics. Demographic characteristics were compared with either a parametric or non-parametric test as adequate. All summed scores were assessed for normality using Shapiro-Wilk test for normality. Categorical variables were compared with chi-square test. Logistic regression analysis was used to model the odds of self-medication by gender and settlement. A *p*-value of < 0.05 was considered as significant. All data analyses were performed using SPSS version 24.0.

## Results

Four hundred and eighty-two (482) questionnaires were received and analyzed from 240 (49.8%) males and 242 (50.2%) females from six states including, Lagos (94), Ebonyi (78), Delta (82), Plateau (77), Borno (76) and Jigawa (75). Majority of the respondents (43%) were between the ages of 18 and 34 years with a mean age of 33.2 ± 12.8 years (95% Confidence Interval [CI] 31.8–34.6) and a median monthly income of 57.5USD (range ≤ 1USD - 1232.9 USD). 112(23.2%) of the respondents specified that they had no income while 20.5% declined disclosure. Of the 482 respondents, 322 (66.8%) had taken antibiotics in the last 6 months (41.5% in the last month and 25.3% in the last six months), while only 68.7% received prescription for them (Table 1). Respondents from the urban area were more likely to have taken antibiotics within the last 6 months than their rural counterparts (Odds ratio [OR] =1.2, 95% CI = 0.8–1.8). Respondents’ settlement category was the only significant demographic predictor (*p* = 0.004) of self-medication found, as urban respondents were more likely to take antibiotics without prescription than rural respondents (OR = 1.9; CI = 1.2–2.8).

**Table 1:** Socio-Demographic characteristics of participants

Variable	Frequency *N* = 482	Percentage (%)
Gender		
Male	240	49.8
Female	242	50.2
Age		
18–24	91	18.9
25–34	116	24.1
35–44	63	13.1
45–54	33	6.8
55–64	16	3.3
65–74	5	1.03
75+	3	0.6
Missing	155	32.2
State		
Borno	76	15.8
Delta	82	17
Ebonyi	78	16.2
Jigawa	75	15.6
Lagos	94	19.5
Plateau	77	16
LGA		
Rural	208	43.2
Urban	274	56.8
Degree		
No schooling	40	8.3
Primary education	37	7.7
Secondary education	167	34.6
Vocational training	49	10.2
Bachelor’s degree	129	26.8
Master’s degree	30	6.2
Doctorate degree	2	0.4
Non-formal education	28	5.8
Last intake of antibiotics		
In the last month	200	41.5
In the last 6 months	122	25.3
In the last year	36	7.5
More than a year ago	48	10
Never	16	3.3
Can’t remember	60	12.4
Received prescription		
Yes	331	68.7
No	151	31.3

**Fig. 1:**
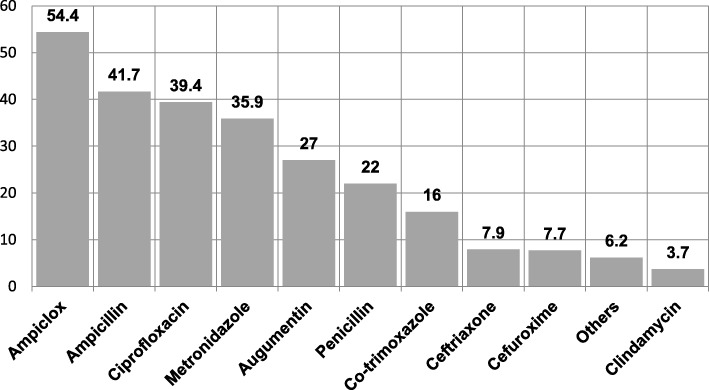
Antibiotics commonly used by the respondents

Majority of the respondents (70.3 and 14.5%) procured the antibiotics from pharmacy store and Chemist, respectively, while 2.1% purchased from hawkers. The most consumed antibiotics were Ampicillin/cloxacillin (54.4%), Ampicillin (41.7%) and Ciprofloxacin (39.4%) while the least consumed was Clindamycin (3.2%) Fig. 1. Other antimicrobials listed by respondents that make up 6.2% included, Tetracycline, Amoxicillin, Gentamycin, Azithromycin, Fluconazole, Sulphonamide, Chloramphenicol, and Cefixime, while one person mentioned taking local herbal mixtures.

Evaluation of knowledge of the respondents revealed that, only about 8.3% of the respondents scored 75% and above and were classified as having good knowledge of AMR. Forty-one (8.5%) participants scored zero point while a few (0.4%) answered correctly to all eight questions and obtained the maximum score. The modal knowledge assessment score was 5. There was no association between gender and the level of knowledge (*p* = 0.13) although males had higher level of knowledge than the females. Comparison of knowledge score across the different states was significant with respondents from Ebonyi (17.9%), Delta (13.4%) and Plateau (10.4%) states more likely to have good knowledge of AMR than participants from Jigawa (5.3%), Borno (2.6%) and Lagos (1.1%) states (X^2^ = 53.22, *p* < 0.0001). Knowledge level was also significantly associated with level of education (Table 2). Respondents in the urban area seemed to have a better knowledge score than the rural respondents. However, except for Borno State, this was not statistically significant both within and across states (Tables 2 and 3).

**Table 2:** Distribution of knowledge score among respondents

Variable	Knowledge Score			Chi-squared (X^2^)	*P*-value
	Poor (%)	Fair (%)	Good (%)		
Gender					
Male	76 (31.7)	138 (57.5)	26 (10.8)	4.07	0.13
Female	83 (34.3)	45 (59.9)	14 (5.8)		
Total	159 (33.0)	283 (58.7)	40 (8.3)		
Settlement category					
Rural	80 (38.5)	114 (54.8)	14 (6.7)	5.36	0.069
Urban	79 (28.8)	169 (61.7)	26 (9.5)		
State					
Borno	36 (47.4)	38 (50.0)	2 (2.6)	53.22	< 0.0001
Delta	19 (23.2)	52 (63.4)	11 (13.4)		
Ebonyi	9 (11.5)	55 (70.5)	14 (17.9)		
Jigawa	20 (26.7)	51 (68.0)	4 (5.3)		
Lagos	41 (43.6)	52 (55.3)	1 (1.1)		
Plateau	34 (44.2)	35 (45.5)	8 (10.4)		
Education					
No schooling completed	20 (50.0)	18 (45.0)	2 (5.0)	39.156	< 0.0001
Primary Education	19 (51.4)	18 (48.6)	0 (0)		
Secondary Education	51 (30.5)	103 (61.7)	13 (7.8)		
Vocational Training	24 (49.0)	23 (46.9)	2 (4.1)		
Bachelor’s degree	35 (19.4)	88 (68.2)	16 (12.4)		
Master’s degree	7 (23.3)	17 (56.7)	6 (20.0)		
Doctorate degree	1 (50.0)	1 (50.0)	0 (0)		
Non-formal Education	12 (42.9)	15 (53.6)	1 (3.6)

**Table 3:** Distribution of Knowledge score according to settlement category within States

State			Knowledge score			Total	X^2^	***P***. value
			Poor (%)	Fair (%)	Good (%)			
**Borno**	Area	Rural	25 (71.4)	10 (28.6)	0 (0)	35	15.59	< 0.001
		Urban	11 (26.8)	28 (68.3)	2 (4.9)	41		
	**Total**		**36 (47.4)**	**38 (50.0)**	**2 (2.6)**	**76**		
**Delta**	Area	Rural	10 (27.8)	20 (55.6)	6 (16.6)	36	1.72	0.423
		Urban	9 (19.6)	32 (69.6)	5 (10.9)	46		
	**Total**		**19 (23.2)**	**52 (63.4)**	**11 (13.4)**	**82**		
**Ebonyi**	Area	Rural	4 (12.1)	23 (69.7)	6 (18.2)	33	0.024	0.988
		Urban	5 (11.1)	32 (71.1)	8 (17.8)	45		
	**Total**		**9 (11.5)**	**55 (70.5)**	**14 (17.9)**	**78**		
**Jigawa**	Area	Rural	11 (34.4)	20 (62.5)	1 (3.1)	32	2.0	0.367
		Urban	9 (20.9)	31 (72.1)	3 (7.0)	43		
	**Total**		**20 (26.7)**	**51 (68.0)**	**4 (5.3)**	**75**		
**Lagos**	Area	Rural	21 (46.7)	24 (53.3)	0 (0)	45	1.16	0.559
		Urban	20 (40.8)	28 (57.1)	1 (2.0)	49		
	**Total**		**41 (43.6)**	**52 (55.3)**	**1 (1.1)**	**94**		
**Plateau**	Area	Rural	9 (33.3)	17 (63.0)	1 (3.7)	27	5.696	0.069
		Urban	25 (50.0)	18 (36.0)	7 (14.0)	50		
	Total		**34 (44.2)**	**35 (45.5)**	**8 (10.4)**	**77**		
**Total**	Area	Rural	80 (38.5)	114 (54.8)	14 (6.7)	208	5.358	0.069
		Urban	79 (28.8)	169 (61.7)	26 (9.5)	274		
	**Total**		**159 (33.0)**	**283 (58.7)**	**40 (8.3)**	**482**		

One hundred and twenty-six (26.1%) respondents across the six states included in the survey admitted that they would stop taking antibiotics when they feel better as opposed to when they have completed the required dosage. However, the response to this question varied among different states, with participants from Lagos (41.5%), Jigawa (32.0%) and Borno (30.3%) more likely to stop taking antibiotics when they feel better (X^2^ = 49.36, *p* < 0.0001). A substantial number of the respondents (26.6 and 37.6%) stated that they would use antibiotics that were prescribed for a relation for a similar illness or would buy antibiotics used in treatment of a previous illness respectively.

**Fig. 2:**
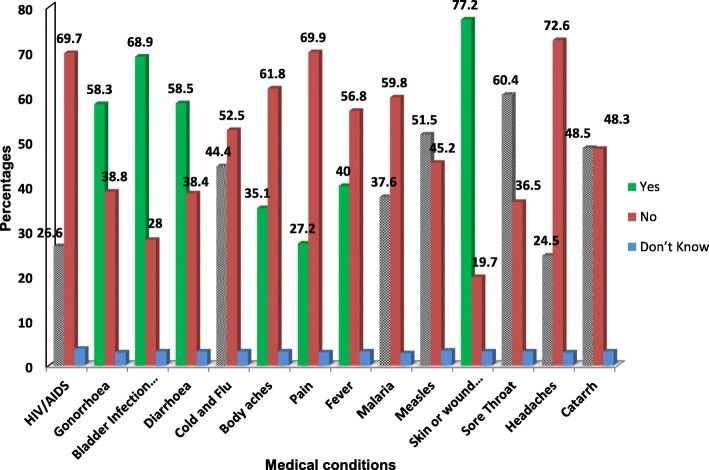
Participants’ response to conditions that can be treated with antibiotics. The grey bars represent common misconception of the use of antibiotics for viral infections

**Fig. 3:**
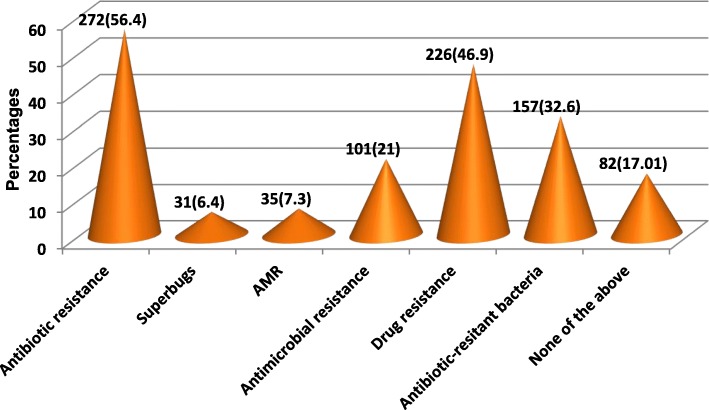
Respondents familiarity with terms used for drug resistance

**Fig. 4:**
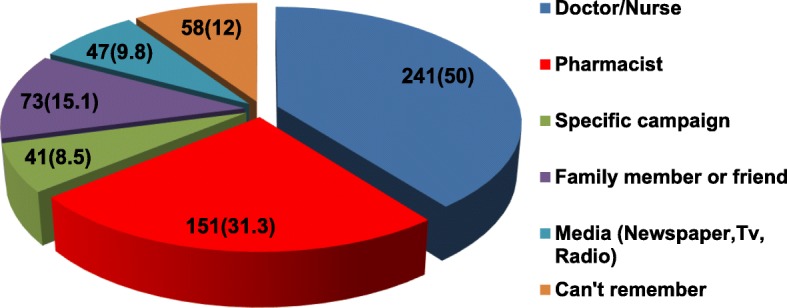
Source of information on Antimicrobial resistance

Figure 2 shows the common conditions treated with antibiotics. According to the respondents, the most common indication for taking antibiotics is skin/ wound infections (77.2%) and bladder infection (68.9%). Nonetheless, 234 (48.5%), 214 (44.4%) and 248 (51.5%) respondents erroneously identified catarrh, cold and flu and measles, respectively, as conditions requiring antibiotics. The vast majority of the respondents were familiar with the term “antibiotic resistance” (56.45%) and “drug resistance” (46.9%) while only 6.4% have heard of the term superbug (Fig. 3). When asked for the source of information on AMR, majority mentioned health practitioners (Doctors, Nurses and Pharmacist) and family members (Fig. 4).

Questions on the perception and attitude towards antimicrobial resistance were answered as follows:

- 86% (417) agreed that people should use antibiotics only when they are prescribed by a doctor.
- 76.4% (368) accept that people should not keep antibiotics and use them later for other illnesses.
- 91.0% (439) endorsed regular hand washing to prevent the spread of AMR.
- 76.7% (370) believed that antibiotic resistance is one of the biggest problems the world is facing.
- 79.5% (383) agreed that responsible intake of antibiotics is everyone’s responsibility.
- 77.4% (373) believed that they are not at risk of getting an antibiotic-resistant infection as long as they take their antibiotics correctly.

**Fig. 5:**
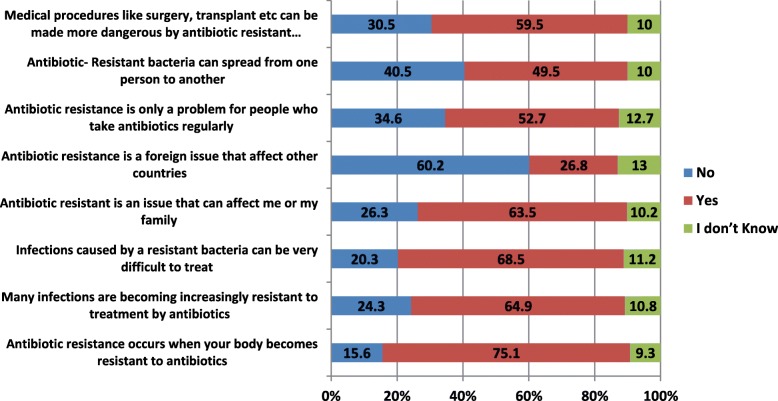
Overview of Responses to knowledge questions

Figure 5 details the responses of the participants to the knowledge questions. 75.1% of the respondents believe that antibiotic resistance occurs when the body becomes resistant to antibiotics instead of when a bacterium becomes resistant to antibiotics. In the same vein, 52.7% of the respondents were of the notion that antibiotic resistance is only a problem for people who take antibiotics regularly while 40.7% did not know that antibiotic-resistant bacteria could spread from person to person. 37.1% of the respondents were not aware of the role of antibiotics use in Agriculture in the spread of AMR and 12.2% believe that it’s not related. However, 76.6% think that there’s nothing they can do to stop the spread of antimicrobial resistance.

## Discussion

In Nigeria, prescription monitoring is poorly conducted and prescription drugs, including, antimicrobials are routinely sold over the counter in pharmacies and by patent proprietary medicines vendors [14]. Consequently, there is risk of increase in antimicrobial resistance in the country. This has been worsened by the lack of confidence in the public health sector due to drug shortages and poor medicine accessibility. The present study adapted and utilized a modified version of the WHO questionnaire on antimicrobial resistance. About two-thirds of the respondents (66.8%) have taken antibiotics in the last 6 months with 41.5% taking antibiotics within a month to the survey. This result is slightly lower than 56.5% monthly consumption rate reported among consumers patronizing pharmacies in Jos [13] and 51.4% reported among non-medical university students in Uyo, Nigeria [15]. Moreover, when compared with the 2015 survey report by WHO for Nigeria (72% antibiotics consumption within six months) there seems to be a slight reduction after four years [7]. Unlike similar studies conducted in the United States, Italy and Namibia [16–18], with a higher level of education among participants, our respondents reported a lower level of education with over 50 % of them (66.6%) without a degree. Respondents with no schooling completed were more likely to have poor knowledge of AMR and this corresponds with reports from other countries [7, 16–19].

The median monthly income of 57.5 USD recorded in this study shows that majority of the respondents earn approximately 1.9USD per day. This value will probably be lower if the 23.2% of the respondents who indicated that they had no income were taken into account. This finding supports the report by the Nigeria Center for Disease Control (NCDC), stating that majority of the Nigerian populace survive on or under 3.10USD per day. The report of that AMR situation analysis which was done in collaboration with the Federal Ministry of Agriculture, Environment and Health went further to identify poverty as the major contributor to the health challenges in Nigeria [14]. The main drivers of antimicrobial use and misuse are patients, farmers and the general populace who demand antimicrobials for real or presumed infections and procure them from unauthorized sources even when they are not prescribed.

Self-medication is a common phenomenon in both developed and developing countries with rates ranging from 3 to 75% [20, 21]. The prevalence of self-medication recorded in this study (31.1%) was similar to previous reports within and outside Nigeria [13, 22]. On the other hand, our result was slightly higher than the 23% self-medication rate reported by WHO for Nigeria [7]. The apparent difference in the results could possibly mirror an increase in self-medication or may be as a result of broader coverage in this study. A study in Namibia reported lower rate of self-medication (15%) with 80% of participants completing the antibiotics course [16]. Similarly, a survey conducted in Italy involving 797 university students reported that out of 30% of the respondents who took antibiotics within 6 months to the survey, 94% were prescribed by a doctor and taken in the correct dose and for the proper duration [18]. About one-quarter (26.1%) of respondents across the six states included in this survey would stop taking antibiotics when they feel better as opposed to when they have taken all of them as directed. Even so, previous studies among university students in different parts of Nigeria have reported lower values for adherence to dosage (40–45%) [12, 15].

The very low knowledge level reported in this study (8.3% good knowledge) contradicts higher scores reported by previous studies from different parts of Nigeria [13, 15]. The large discrepancy from previous studies may be attributable to variation in the study population. Whereas these previous studies targeted the learned population, the current study aimed for wider inclusivity to account for the underserved population. Of interest is the fact that respondents from Lagos State had the least level of knowledge of AMR and were more likely to stop taking antibiotics when they feel better (X^2^ = 49.36, *p* < 0.0001). Lagos State, which is the commercial hub of Nigeria, is home to over 17.5 million people [23].

The findings of this study reveal overall deficiency in the knowledge of proper antibiotic use and AMR in the wider Lagos population. The very busy lifestyle and heavy commercial activities in Lagos may have significantly contributed as barrier to awareness dissemination. The result from Borno State on the other hand, came as no surprise because of the security crisis ravaging the state, which has hindered access to educational and health facilities. Except for Borno State where there was statistically significant difference in the knowledge level between urban and the rural community, the rest of the states did not show any difference in the settlements. This goes to show that the overall poor level of knowledge is irrespective of gender and place of abode.

The result of our findings reveals that people are inclined to purchase and use same antibiotics from previous illnesses. Although majority of the respondents correctly identified skin/wound infection (77.2%) and bladder infection (68.9%) as conditions treatable with antibiotics, quite a number of them also selected headaches, body aches, pain including viral infections (cold and flu, Catarrh, Measles and HIV), as indications for antibiotics use. This has exposed a high level of misconceptions amongst the Nigerian public on the indications for the use of antibiotics and the need for health workers to explain the difference between viral and bacterial infections when communicating with patients.

Interestingly, although 76.4% agreed that people should not keep antibiotics and use them later for other illnesses, a substantial number of the respondents (37.6%) categorically stated that they would buy and use antibiotics that were used previously for a similar illness while 26.6% would use leftover drugs from a previous illness to treat a current illness. Similarly, a previous study in Jos, Nigeria reported antibiotics sharing rate of 48% among respondents patronizing four community pharmacy outlets [13]. This buttresses the fact that majority of the people who abuse antibiotics do so because of over the counter availability of these drugs. Hence, if we must win the war against emergence of antimicrobial resistance, the primary target should be effective regulation of pharmacy shops and dispensaries.

The result of the multiple-choice question on familiarity with terms used to describe antimicrobial resistance clearly shows that any awareness campaign targeted at the general public should be done using terms that the people are familiar with and fancy terms like superbug and AMR should be avoided. Worthy of mention is the fact that 17.0% of the respondents have never heard of antimicrobial resistance or any of the terms used for it even though they use antibiotics. Subsequently, this subset of individuals declined answering the AMR knowledge questions as they have absolutely no knowledge and were grouped under those with poor knowledge. In addition, this study revealed that respondents from the urban area were more likely to have taken antibiotics within the last 6 months than their rural counterparts. This indicates that contrary to popular belief, antibiotics consumption maybe more rampant in the urban than rural areas.

Regrettably, specific campaigns (8.5%) and media (9.8%) got the least mention as a source of information on AMR. This finding is interesting because we are in the digital age where media tools, especially television, have become indispensable for awareness campaigns. Clearly, more needs to be done by way of engaging the media as a viable tool for science communication. Perhaps this will ultimately enhance visibility and increase awareness of AMR.

Another point of concern is the fact that 76.6% of the respondents believe that there’s nothing they can do to stop the spread of antibiotic resistance. On the contrary, everyone has a role to play and community engagement is a veritable tool to mitigate the spread of AMR. This underscores the need for proper awareness campaign and education using avenues that will have a wider coverage.

Furthermore, drug misuse also extends to the agricultural sector where antimicrobials are liberally used therapeutically and for growth promotion [14]. In this study, 37.1% of the respondents were not sure of the role of “antibiotics use in agriculture” in the spread of AMR in the human population while 12.2% did not think they were related. Also, 77.4% (373) believed that they were not at risk of getting an antibiotics-resistant infection as long as they took their antibiotics correctly. Unfortunately, this statement is far from correct as resistant strains can be transferred from person to person thereby constituting a threat to even the diligent ones.

One of the major strengths of this study is the ability to evaluate the knowledge and perception of persons at the grassroots about AMR unlike previous studies which have focused on university students and the literate population. The use of local interpreters allowed for the inclusion of the illiterate under-represented fraction of the Nigerian populace. In addition, some other studies have used crowd-sourcing technology [17] which is limited by exclusion of a fundamental part of the population that are not computer literate. This study gives a comprehensive baseline information of the current level of awareness of AMR among the Nigerian populace as well as the common behaviors and practices that fuel emergence of resistance. The use of closed and open-ended questions in this study allowed for exploring the respondents level of understanding and intent.

This study like most questionnaire-based study, faced limitations due to respondent’s bias in which case some of the respondents felt the need to give a “correct” answer instead of a sincere response. Also, selection of one state from each geopolitical zone with a total of six states may limit the robustness of the data generated. Furthermore, quite a handful of the respondents 32.2 and 20.5% declined to disclose their age and monthly income respectively. This restricted our ability to make evocative inferences using age and income.

The none-disclosure of age may be attributed to the fact that some of the participants especially in the rural areas, were not sure of their actual age and also taking into account that some traditional practices of persons in the local community, particularly in the rural areas, restrict them from disclosing their age.

In addition, the insecurity issues in some parts of the country may have contributed to non-disclosure of income.

## Conclusion

This survey presents a national average as well as region/state specific data on AMR awareness drawing out the differences in the findings between states, settlements and educational background. There was an overall poor knowledge and awareness of AMR across the study area and this was irrespective of gender, age, state and settlement category. It is critical that people understand the problem of AMR, and the way in which their behavior affects AMR emergence and the role they can play to change this trend.

## Data Availability

All data generated or analyzed during this study are included in this published article [and its supplementary information files] Original data are available from the corresponding author upon reasonable request.

## Abbreviations

WHO: World health organization
AMR: Antimicrobial resistance
NIMR: Nigerian institute of medical research
SPSS: Statistical package for social sciences
USD: United states dollars
GAP: Global action plan
LGA: Local government area
OR: Odds ratio
CI: Confidence interval
